# A Randomized Controlled Trial Comparing the Effects of Sitagliptin and Glimepiride on Endothelial Function and Metabolic Parameters: Sapporo Athero-Incretin Study 1 (SAIS1)

**DOI:** 10.1371/journal.pone.0164255

**Published:** 2016-10-06

**Authors:** Hiroshi Nomoto, Hideaki Miyoshi, Tomoo Furumoto, Koji Oba, Hiroyuki Tsutsui, Atsushi Inoue, Tatsuya Atsumi, Naoki Manda, Yoshio Kurihara, Shin Aoki, on behalf of SAIS Study Group

**Affiliations:** 1 Division of Rheumatology, Endocrinology and Nephrology, Hokkaido University Graduate School of Medicine, Sapporo, Japan; 2 Department of Cardiovascular Medicine, NTT East Japan Sapporo Hospital, Sapporo, Japan; 3 Department of Cardiovascular Medicine, Hokkaido University Graduate School of Medicine, Sapporo, Japan; 4 Department of Biostatistics, School of Public Health, Graduate School of Medicine, The University of Tokyo, Tokyo, Japan; 5 Interfaculty Initiative in Information Studies, The University of Tokyo, Tokyo, Japan; 6 Japan Community Healthcare and Organization Hokkaido Hospital, Sapporo, Japan; 7 Manda Memorial Hospital, Sapporo, Japan; 8 Kurihara Clinic, Sapporo, Japan; 9 Aoki Clinic, Sapporo, Japan; Florida International University Herbert Wertheim College of Medicine, UNITED STATES

## Abstract

**Objectives:**

The DPP-4 inhibitors are incretin-related drugs that improve hyperglycemia in a glucose-dependent manner and have been reported to exert favorable effects on atherosclerosis. However, it has not been fully elucidated whether DPP-4 inhibitors are able to improve endothelial function in patients with type 2 diabetes. Therefore, we investigated the efficacy of sitagliptin, a DPP-4 inhibitor, on endothelial function and glycemic metabolism compared with that of the sulfonylurea glimepiride.

**Materials and Methods:**

In this multicenter, prospective, randomized parallel-group comparison study, 103 outpatients with type 2 diabetes (aged 59.9 ± 9.9 years with HbA1c levels of 7.5 ± 0.4%) with dietary cure only and/or current metformin treatment were enrolled and randomly assigned to receive sitagliptin or glimepiride therapy once daily for 26 weeks. Flow-mediated dilation (FMD), a comprehensive panel of hemodynamic parameters (Task Force^®^ Monitor), and serum metabolic markers were assessed before and after the treatment.

**Results:**

During the study period, no statistically significant change in %FMD was seen in both groups (sitagliptin, 5.6 to 5.6%; glimepiride, 5.6 to 6.0%). Secretory units of islets in transplantation, TNF-α, adiponectin and biological antioxidant potential significantly improved in the sitagliptin group, and superoxide dismutase also tended to improve in the sitagliptin group, while improvements in HbA1c levels were similar between groups. Cardiac index, blood pressure and most other metabolic parameters were not different.

**Conclusions:**

Regardless of glycemic improvement, early sitagliptin therapy did not affect endothelial function but may provide favorable effects on beta-cell function and on inflammatory and oxidative stress in patients with type 2 diabetes without advanced atherosclerosis.

**Trial Registration:**

UMIN Clinical Trials Registry System UMIN 000004955

## Introduction

Patients with type 2 diabetes are at a markedly higher risk of cardiovascular events compared with those without diabetes [1]. In many large clinical studies, it has been demonstrated that the prevalence of coronary and peripheral artery disease is 2- to 4-fold higher, and even the risk of stoke was 2-fold higher in patients with overt type 2 diabetes [2–4]. Therefore, for this patient population, prevention and improvement of atherosclerosis are as important as maintaining favorable blood glucose control. Recently, endothelial cell dysfunction has been identified as one of the earliest changes in the development of atherosclerosis [5]. Additionally, it has been reported to be an important predictor of cardiovascular events in patients with type 2 diabetes [6, 7]. Moreover, endothelial cell function is used as a therapeutic surrogate parameter of the early phase of atherosclerosis because of its plasticity. Flow-mediated dilation (FMD) of the brachial artery reflects endothelial nitric oxide (NO) bioavailability and is widely used as a marker for early atherosclerosis [8, 9]. Indeed impaired FMD is associated with type 2 diabetes independent of glucose levels and may, in part, explain the increased cardiovascular risk in this patient population [10]. Therefore, it is important that diabetes treatment not only achieves glycemic control but that it also maintains/improves FMD to prevent the development of vascular complications.

Recently, incretin drugs such as DPP-4 inhibitors and GLP-1 mimetics have been widely approved for the treatment of type 2 diabetes. These drugs not only improve glycated hemoglobin A1c (HbA1c) and glycemic control, but are also expected to have anti-atherosclerotic and beta-cell protective effects. Indeed, DPP-4 inhibitors have been reported to possess protective effects on atherosclerosis in some animal models and in vitro experiments; however, some large clinical studies focusing on cardiovascular events could not verify the superiority of DPP-4 inhibitors [11–13]. To investigate whether incretin drugs including DPP-4 inhibitors have an effect on the earlier phase of atherosclerosis or not, some clinical trials have been conducted using FMD, but the results have been inconsistent [14–16]. One of the possible reasons for theses varying results may be the difficulty associated with consistently assessing FMD because of technical and environmental factors. We previously reported that one of the incretin mimetics, liraglutide did not improve FMD in type 2 diabetes in spite of its potent hypoglycemic effect, anti-atherosclerotic and anti-oxidative effects [17]. In this trial, we aimed to assess the effects of sitagliptin, a DPP-4 inhibitor, on endothelial function in patients with type 2 diabetes using a multicenter, prospective, randomized parallel-group comparison study design.

## Materials and Methods

### Study population

We enrolled 103 subjects with type 2 diabetes and adequately controlled blood pressure and plasma lipids from 9 medical service units located in Sapporo City (SAIS Study Group). We included patients with type 2 diabetes who were treated with or without metformin, were between 20 and 75 years of age, and had inadequate glucose control (defined as HbA1c between 6.9 and 8.4%). We excluded patients who were diagnosed with atherosclerotic diseases (angina, myocardial infarction, cerebral infarction and peripheral arterial disease), were currently receiving insulin therapy, were pregnant women, had a persistent elevation of their serum transaminase levels or had renal dysfunction.

### Protocol

This was a multicenter, open-labeled prospective randomized, parallel-group comparison study. Following enrollment, all individuals visited the Hokkaido University Hospital for measurement of a comprehensive panel of hemodynamic parameters (Task Force^®^ Monitor), and serum metabolic markers. At the same time, FMD was performed by a well-trained technician who was blinded to the study to minimize the introduction of potential confounding variables given the sensitive nature of the FMD analysis. The primary endpoint of the study was the extent of change in FMD. The sample size was calculated using the assumption that sitagliptin and glimepiride would improve FMD by at least 1.0% (SD = 2.0%) and 0.1% (SD = 1.0%), respectively, based on previous studies using glimepiride and pioglitazone [18], or continuous infusion of GLP-1 [19]. It was determined that 50 patients were needed for each group to detect a significant difference with an 80% power and statistical significance of 5%, assuming unequal variance between groups, based on the two-sample *t*-test. Secondary endpoints were changes in metabolic parameters and surrogate markers of β cell function.

Patients were randomly assigned to receive once daily sitagliptin (50–100 mg/day) or glimepiride (0.5–2.0 mg/day) according to their age, body mass index and results of FMD using a computer software. All patients were encouraged to continue diet and exercise therapy during the study. Treatment was performed at each respective medical care center for 24 weeks; then, endothelial function and serum biomarkers were measured again at the end of the study at Hokkaido University Hospital using the same parameters used at baseline. The subject enrollment period was from March 2011 to 30 September 2013. The last subject completed the study in May 2014.

### Flow-mediated dilatation and Taskforce Monitor

Endothelial function was evaluated using FMD of the brachial artery, according to published guidelines [20–22]. Briefly, the study was performed in the morning following an overnight fast and before taking any medications. Participants were asked to abstain from smoking, consuming caffeine and antioxidant vitamins on the day of the evaluations. They received only drinking water prior to the FMD assessment. Patients were asked to remain in supine position for at least 15 min in a quiet, temperature-controlled room (23°C to 26°C). Then, baseline FMD was measured using the brachial artery of their right arm. After 5 min of suprasystolic compression (50 mmHg over the systolic blood pressure) on the right forearm, the cuff was deflated and FMD was measured again. The %FMD is expressed as percentage change from baseline to peak dilatation. All FMD measurements were made at a single location (Hokkaido University Hospital) by the same well-qualified technician that was blinded to the treatment groups.

After FMD measurement, beat-to-beat continuous finger BP, low frequency/high frequency ratio of the R-R interval (LF/HF-RRI), cardiac index and total peripheral resistance index (TPRI) were measured noninvasively using a Task Force Monitor system (CNSystem, Austria).

### Biochemical analysis

For fasting serum analysis, collected blood samples were immediately placed on ice, centrifuged at 4°C, and the isolated supernatant was frozen until measurement. LDL-cholesterol levels were calculated using the Friedewald formula. Plasma adiponectin, high-sensitivity C-reactive protein (hs-CRP), glucagon, TNF-α, total PAI-1, NT-proBNP, proinsulin and super oxide dismutase (SOD) were measured by latex agglutination, nephelometry, radioimmunoassay, ELISA, latex photometric immunoassay, electrochemiluminescence immunoassay, and improved nitrite ion method, respectively (SRL, Inc., Tokyo, Japan).

### Evaluation of relevant factors

Secretory units of islets in transplantation (SUIT), proinsulin/insulin ratio and C-peptide index were calculated for to assess β-cell function. SUIT and C-peptide index were assessed using the following formulas: SUIT = [CPR (ng/ml) × 1500] / [Fasting plasma glucose (mg/dl)– 61.7], C-peptide index = [CPR (ng/ml) / Fasting plasma glucose (mg/dl)] × 100.

### Derivatives of reactive oxygen metabolites (d-ROM) and biological anti-oxidant potential (BAP) test

According to previous reports [23], the level of d-ROM was measured as an index of production of reactive oxygen species and BAP was measured as an index of anti-oxidant potential using a free radical elective evaluator (the Free system, Diacron, Italy). Briefly, 20 μl of serum were mixed with acetic acid buffer (pH 4.8) in a pipet to stabilize the hydrogen ion concentration. Bivalent and trivalent iron was separated from serum proteins in an acidified medium. Blood proteins were placed in the acetic acid buffer and processed to catalyze the production free alkoxy and peroxy radicals from hydroperoxide groups in the serum. Then, these mixtures were transferred into cuvettes containing the colorless chromogen N, N-diethyl-p-phenylenediamine which turns to magenta if oxidized by free radicals into radical cations. The intensity of the magenta color reflects the concentration of hydroperoxides in the serum. The cuvettes were incubated for 5 min at 37°C, and the intensity of the magenta color of the cuvettes was measured by photometry (505 mm) after centrifuging for 1 min to determine the concentration of hydroperoxide. The values of hydroperoxide are shown as arbitrary units (U. CARR) [24].

The BAP was simultaneously measured. The trivalent FeCl_3_ salt turns red because of the anti-oxidant action of trivalent Fe^2+^ ions. In theory, the anti-oxidant potential of serum can be evaluated by measuring the degree of decolorization using a photometer. In practice, the amount of trivalent iron from 10 μl of serum that is deoxidized in 5 min was measured in units of μmol/l [24].

### Statistical Analysis

Results are expressed as means and medians and range. Differences of baseline characteristics between both groups were assessed by Welch’s *t*-test or Mann-Whitney U test for continuous variables and Chi-square test for categorical variables. The Kolmogorov-Smirnov test for normality was used to determine the appropriate statistical test for the continuous variables. The primary endpoint was analyzed based on the intention-to-treat principle. As the primary analysis, the effects of sitagliptin compared with glimepiride on FMD were assessed by ANCOVA adjusted for baseline FMD. Multiple imputations were used to handle the missing outcomes (e.g., FMD after 26 weeks). To impute the missing data, we used the predictive mean matching method, including variables potentially related to the fact that the FMD was missing and also that variables correlated with FMD. The number of imputations was repeated by 100 times.

For the secondary endpoints, mean changes between baseline and post-treatment of endothelial and metabolic parameters in both groups were analyzed descriptively in the complete case population. We also employed paired t-test or Wilcoxon signed test without the adjustment of the multiplicities because these secondary analyses were exploratory results. The relationship between changes in %FMD and other metabolic variables, such as body mass index (BMI), HbA1c, cholesterol and blood pressure, were assessed using Spearman’s rank-correlation analysis. A p-value <0.05 was considered statistically significant. Data were analyzed using SAS version 9.4 (SAS Institute Inc., Cary, NC, USA.) and Ekuseru-Toukei 2012 (Social Survey Research Information, Tokyo, Japan).

### Ethics statement

This study protocol was reviewed and approved by the institutional review board of Hokkaido University and written consent was obtained from all participants. This study has been registered in the UMIN Clinical Trials Registry System under the identifier UMIN 000004955.

## Results

### Baseline characteristics

**Fig 1:**
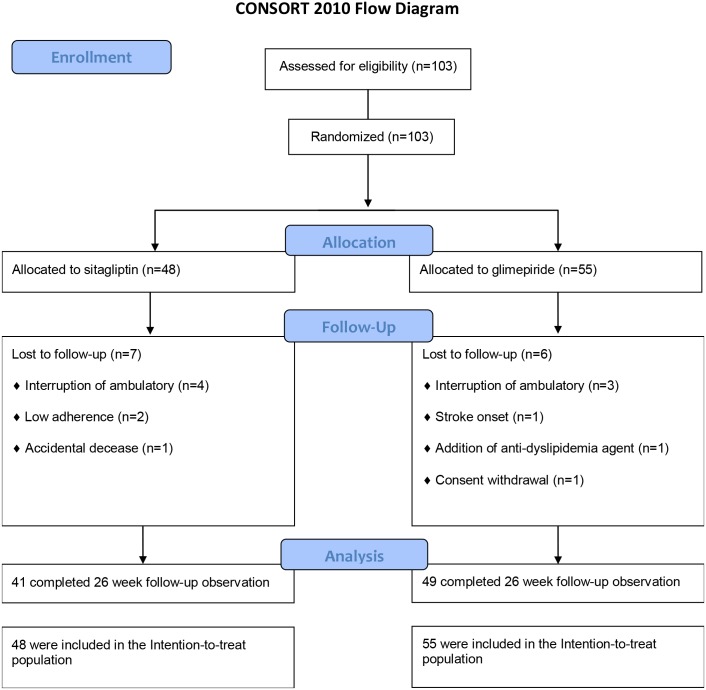
CONSORT flow diagram.

In total, 103 individuals were enrolled and underwent first examination. All of them were randomized to each group, and none was excluded. At the end of the study, 13 participants did not undergo the final examination for the following reasons: seven patients for interruption of ambulatory visit, two patients for low adherence, and one each for the addition of an anti-dyslipidemia agent, stroke onset, accidental decease and consent withdrawal (Fig 1). The participants consisted of 62 men and 41 women with a mean age of 59.9 ± 9.9 years and mean HbA1c levels of 7.5 ± 0.5%. Other baseline characteristics of each group are shown in Table 1. There were no differences between the two groups in BMI, blood pressure, biological parameters, prevalence of current smoking, complications of diabetes and proportion of oral hypoglycemic agents, renin-angiotensin-system blockers and statins. Sitagliptin and glimepiride were well tolerated throughout the study, except for the discontinuations described above.

**Table 1:** Clinical characteristics of the study population.

Variables	Sitagliptin (n = 48)	Glimepiride (n = 55)	*P* value
**Age (years)**	62 (35–80)	60 (36–60)	0.86*
**Male sex (%)**	68.8	52.7	0.10
**Body mass index (kg/m^2^)**	25.7 ± 3.9	25.2 ± 3.5	0.48
**Flow mediated dilatation (%)**	5.6 ± 2.8	5.6 ± 2.2	0.91
**FPG (mM/L)**	8.2 ± 1.4	8.0 ± 1.7	0.40
**Hemoglobin A1c (%)**	7.4 (6.7–8.4)	7.4 (7.5–8.6)	0.84*
**SBP (mmHg)**	130.0 ± 14.7	133.0 ± 17.6	0.35
**DBP (mmHg)**	77.4 ± 10.1	77.6 ± 9.4	0.92
**LDL-cholesterol (mg/dl)**	101.4 ± 25.0	105.3 ± 26.7	0.45
**Current smokers (%)**	31.3	29.1	0.97
**Hypertension (%)**	54.2	61.8	0.73
**Dyslipidemia (%)**	68.8	74.5	0.81
**Angiotensin-converting enzyme inhibitors/ angiotensin II receptor blockers (%)**	41.7	47.3	0.85
**Statin (%)**	41.7	54.5	0.43
**Metformin (%)**	60.4	50.9	0.63
**Diabetic nephropathy (%)**	20.8	32.7	0.40

FPG, fasting blood glucose; SBP, systolic blood pressure; DBP, diastolic blood pressure; DPP-4, dipeptidyl peptidase-4. Values are mean ± SD or median (range). *P* value of liraglutide vs glargine treated groups.

* Mann–Whitney U test was applied to the factors age and HbA1c.

### Endothelial function and atherosclerosis

The average baseline FMD measurements were 5.6% and 5.6% for sitagliptin and glimepiride, respectively. As shown in Table 2, the increment in %FMD after treatment seemed to be greater with glimepiride than with sitagliptin, but not significant in either group. Even after adjusting for baseline FMD measurements, the lack of significant differences between groups remained (Table 2). There was no obvious relationship between the changes in FMD and HbA1c (Table 3), but a positive correlation was observed between ΔFMD and baseline BMI in the sitagliptin group (S3 Table). Other cardiovascular parameters, such as cSBP, augmentation index, cardiac index and TPRI, remained unchanged (Table 4).

**Table 2:** Comparison of changes in %FMD before and after treatment in each group.

	Sitagliptin (n = 48)	Glimepiride (n = 55)	*P* value
**FMD (%)**			
**Baseline**	5.6 ± 2.8	5.6 ± 2.2	
**26 weeks**	5.6 ± 2.8	6.0 ± 2.3	
**ΔFMD (%)**	0.002 (-3.89 to 3.89)	0.43 (-3.57 to 4.44)	
**Comparison of ΔFMD in ANCOVA**	-0.41 (-1.20 to 0.37)	reference	0.303

Values are expressed as mean ± SD or the least square means (95% CI). The least square means were calculated by ANCOVA adjusted for baseline FMD. FMD, flow-mediated dilation; ANCOVA, analysis of covariance; CI, confidence interval

**Table 3:** Relationship between the changes in %FMD and other metabolic parameters pre- and post-treatment with sitagliptin or glimepiride.

Variables	r	*P* value for the Spearman’s rank-correction	r	*P* value for the Spearman’s rank-correction
Medication	Sitagliptin		Glimepiride	
**BMI**	0.189	0.238	-0.056	0.701
**HbA1c**	0.008	0.962	0.073	0.617
**SBP**	-0.273	0.084	-0.103	0.481
**DBP**	-0.165	0.304	-0.113	0.439
**HDL-cholesterol**	-0.067	0.677	0.035	0.809
**LDL-cholesterol**	-0.086	0.618	0.061	0.679

FMD, flow-mediated dilation; BMI, body mass index; SBP, systolic blood pressure; DBP, diastolic blood pressure.

**Table 4:** Comparison of the effects on endothelial function and clinical and biochemical parameters between sitagliptin and glimepiride.

Variables	Sitagliptin (n = 41)		Glimepiride (n = 49)	
	Mean change from baseline (95% CI)	*P* value	Mean change from baseline (95% CI)	*P* value
**BMI (kg/m**^**2**^**)**	0.01 (-0.17 to 0.20)	0.873	0.31 (0.17 to 0.44)	<0.001
**SBP (mmHg)**	3.1 (-0.6 to 6.8)	0.098	3.0 (-1.3 to 7.2)	0.165
**DBP (mmHg)**	0.4 (-1.6 to 2.5)	0.683	0.9 (-1.0 to 2.9)	0.341
	**Cardiovascular functions**			
**cSBP (mmHg)**	3.8 (-0.5 to 8.0)	0.083	1.6 (-4.7 to 7.8)	0.623
^†^**Augmentation index**	-13.0 (3.01 to 17.0)	0.083	0.0 (-12.0 to 12.0)	0.692
*Cardiac index	-0.05 (-0.17 to 0.07)	0.404	0.10 (-0.03 to 0.22)	0.133
*^†^**TPRI**	67 (-739 to 1444)	0.523	-21 (-1070 to 274)	0.642
**^†^**LF/HF ratio of RRI**	0.07 (-2.60 to 4.17)	0.446	0.30 (-0.90 to 3.53)	0.026
	**Biochemical parameters**			
**FPG (mM/l)**	-0.39 (-0.70 to -0.09)	0.014	-0.77 (-1.15 to -0.38)	<0.001
^†^**Hemoglobin A1c (%)**	-0.7 (-1.7 to 0.2)	<0.001	-0.7 (-1.3 to -0.2)	<0.001
^†^**IRI (μU/ml)**	-4.3 (-5.5 to 7.1)	0.285	-2.5 (-4.0 to 3.9)	0.796
**CPR (ng/ml)**	0.10 (-0.05 to 0.25)	0.197	0.05 (-0.10 to 0.19)	0.503
^†^**Proinsulin / IRI ratio**	-6.3 (-52.7 to 28.0)	0.193	-1.4 (-39.5 to 27.6)	0.972
^†^**C-peptide index**	0.13 (-0.39 to 0.47)	0.005	0.18 (-0.24 to 0.66)	<0.001
^†^**SUIT**	6.1 (-9.3 to 17.0)	<0.001	9.7 (-2.7 to 35.3)	<0.001
^†^**Glucagon (pg/ml)**	-5.0 (-41.0 to 33.0)	0.042	-1.0 (-33.0 to 31.0)	0.626
**LDL-cholesterol (mg/dl)**	10.7 (4.5 to 16.8)	0.001	2.1 (-3.6 to 7.9)	0.461
^†^**HDL-cholesterol (mg/dl)**	1.0 (-5.0 to 13.0)	0.020	-2.0 (-11.0 to 12.0)	0.042
^†^**Triglyceride (mg/dl)**	1.0 (-169.0 to 35.0)	0.364	-3.0 (-46.0 to 45.0)	0.554
^†^**Adiponectin (μg/ml)**	0.26 (-0.28 to 2.72)	<0.001	0.13 (-0.85 to 0.92)	0.162
^†^**SOD activity (U/ml)**	0.2 (-0.9 to 1.3)	0.061	0.0 (-0.8 to 0.8)	0.910
^†^**Total PAI-1 (ng/ml)**	-1.0 (-17.0 to 17.0)	0.878	-1.0 (-20.0 to 16.0)	0.409
^†^**NT-proBNP (pg/ml)**	-1.0 (-46.0 to 42.0)	0.754	-2.0 (-43.0 to 42.0)	1.000
^†^**TNF-α (pg/ml)**	-0.2 (-1.2 to 0.6)	0.045	0.0 (-0.5 to 0.7)	0.847
**log hsCRP (ng/ml)**	-0.01 (-0.19 to 0.17)	0.884	0.05 (-0.11 to 0.20)	0.552
^†^**albuminuria (g/g.Cre)**	0.0 (-65.4 to 99.1)	0.357	0.0 (-64.0 to 18.4)	0.601
	**Oxidative stress**			
**d-ROMs**	-1.0 (-34.6 to 32.6)	0.954	20.6 (-0.87 to 0.70)	0.064
^†^**BAP**	238 (-805 to 3411)	0.041	183 (-1563 to 1226)	0.941

* Data were obtained in 87 patients (Sitagliptin N = 40, Glimepiride N = 47).

** Data were obtained in 82 patients (Sitagliptin N = 36, Glimepiride N = 46).

BMI, body mass index; SBP, systolic blood pressure; DBP, diastolic blood pressure; FMD, flow-mediated dilatation; cSBP, centric systolic blood pressure; TPRI, total peripheral resistance index; LF, low frequency; HF, high frequency; RRI, R-R interval; FPG, fasting blood glucose; SOD, superoxide dismutase; PAI-1, plasminogen activator inhibitor-1; NT-proBNP, N terminal prohormone of brain natriuretic peptide; TNF-α, tumor necrosis factor alpha; hsCRP, high-sensitivity C-reactive protein; d-ROMs, reactive oxygen metabolites-derived compounds; BAP, biological antioxidant potential. Values are mean or median change from baseline mean (95% CI).

^†^ Values were analyzed using the Wilcoxon signed rank test because normality was rejected for these variables.

### Glycemic control and metabolic factors

After 6 months of sitagliptin or glimepiride treatment, HbA1c levels were similarly improved in both groups, although a reduction of FPG was greater in the glimepiride group than in the sitagliptin group (p<0.01) (Table 4). In the glimepiride group, HbA1c levels improved similarly regardless of the baseline BMI. However, there was a positive correlation between baseline BMI and change in HbA1c levels in the sitagliptin group (S3 Table). The beta-cell function, assessed by SUIT and C-peptide index, was significantly increased in both groups, although the other index, proinsulin/insulin ratio showed a slight, but not significant, improvement in the sitagliptin group. The serum glucagon level was also reduced in the sitagliptin group (p = 0.04).

Sitagliptin treatment significantly improved the serum adiponectin levels accompanied by the elevation of HDL-C and even LDL-C (p<0.001, p = 0.02 and p = 0.001, respectively). Moreover, there was a significant negative correlation between baseline BMI and changes of adiponectin during the sitagliptin treatment (S3 Table). In regard to inflammatory markers, TNF-α significantly decreased in the sitagliptin group only (p = 0.05), but other inflammatory biomarkers, such as hsCRP, 8-OHdG and d-ROMs did not show significant changes. Sitagliptin treatment also increased anti-inflammatory and anti-oxidant factors such as SOD and BAP (p = 0.06, p = 0.04) during the study period. There were no obvious correlations between the degree of change in %FMD and those of confounding factors, such as BMI, blood pressure or lipid profiles, in both groups (Table 3).

## Discussion

Incretin drugs, including DPP-4 inhibitors, have been reported to exert a protective effect against atherosclerosis by mediation of several pathways; upregulation of the activity and protein expression of endothelial NO synthase; prevention of reactive oxygen species-induced cell senescence in endothelial cell lines [25, 26]; attenuation of TNF-α mediated induction of PAI-1 expression [27]; intracellular adhesion molecule-1 and vascular cell adhesion molecule-1 [28]; inhibition of monocyte adhesion [29] and proliferation of smooth muscle cells [30]. A recent study showed that after 104 weeks of sitagliptin treatment, intima-media thickness in type 2 diabetes mellitus patients was significantly improved [31]. This desirable effect was thought to be partially derived from sitagliptin-induced GLP-1 signaling, which inhibits macrophage accumulation and inflammation, and suppresses macrophage-related inflammation and DPP-4-induced smooth muscle proliferation [31]. GLP-1 mediates pathways through the GLP-1 receptor in atherosclerosis, which was discussed, but it is not clear if the endothelium contains the GLP-1 receptor. A recent study demonstrated that vascular smooth muscle, but not endothelial, cells contain the GLP-1 receptor [32]. Regarding endothelial function, assessed by FMD, our study demonstrated that 26-week sitagliptin treatment for patients with type 2 diabetes did not affect %FMD. Nevertheless, some previous clinical studies have reported on these beneficial effects on endothelial cell function [14, 15]. It has also been reported that calculated FMD is a sensitive parameter that is easily confounded by many factors such as patients’ background (heart rate, sex, age, obesity and smoking) [33], conditions (air temperature, mental/physical stress) [34–36], and medications such as angiotensin II receptor blockers [37], statins [38] and some types of anti-hypoglycemic agents [18, 39]. In this study, there were no significant differences in both groups for any of these confounding factors. Additionally, adjusted ΔFMD which was calculated based on baseline FMD values, yielded similar results in both groups. Moreover all FMD measurements were done by the same well-trained individual under controlled conditions. In the context of the present study, our results clearly demonstrated that sitagliptin did not improve endothelial function measured by %FMD even when compared with individuals treated with glimepiride. It has also been reported that changes of glucose variability do affected the results of FMD in some hypoglycemic agents [16, 40]. In our study, glycemic control was similarly improved in both groups and there was no obvious correlation between the improvement of HbA1c and ΔFMD (Table 3). In the sitagliptin group, we observed that the higher the BMI, the lower the improvement of HbA1c. Similar findings were reported in a previous report [41]. Furthermore, there was a negative correlation between baseline BMI and changes in adiponectin. Nevertheless, improvement of %FMD was greater in obese patients (S3 Table). According to the above results, sitagliptin may, in part, have a somewhat preferable effect on FMD independent of the improvement of HbA1c and adiponectin levels in obese subjects.

Secondary endpoint data from our study suggest possible beneficial effects of sitagliptin on lipid metabolism and inflammatory responses. DPP-4 inhibitors have been verified to improve lipid metabolism according to incremental serum adiponectin levels [42]. While exogenic administration of adiponectin was reported to increase antioxidant factors including SOD [43], we observed a significant increase of serum adiponectin levels with sitagliptin treatment. Not only HDL-C, but also LDL-C levels were significantly elevated without any changes in body weight. We cannot exclude that this induction of LDL increment may partially affect the lack of effect on endothelial function seen in our study. Our study also revealed that sitagliptin treatment reduced TNF-α and increased anti-inflammatory and anti-oxidative responses such as SOD and BAP. TNF-α is a well-known inflammatory cytokine involved in the onset as well as the progression of atherosclerosis that induces the expression of transcriptional factors, such as the nuclear factor-κB. TNF-α has been reported to decrease endothelial dilatation leading to dysfunction of the endothelium [44] and cause apoptosis of the endothelial cells through dephosphorylation of protein kinase B [45]. Moreover, excessive inflammatory cytokines, including TNF-α are also related to pancreatic beta-cell apoptosis [46]. Regarding β-cell protection, significant improvement was observed after both sitagliptin and glimepiride treatments, as assessed by SUIT and C-peptide index, which were the biomarkers of β-cell function. Many basic studies have demonstrated a protective effect of DPP-4 inhibitors on pancreatic β-cells, including reduction of apoptosis [47, 48], for which one of the possible protective pathways is the antioxidant response [49]. Pancreatic β-cells are sensitive to oxidative stresses given their relatively low expression of anti-oxidant enzymes such as SOD, catalase and glutathione peroxidase [50]. In the current study, the BAP level, which quantifies the power of total antioxidant stress activity, was significantly increased with sitagliptin treatment. Both the reduction of inflammatory factors and incremental antioxidant ability may, in part, contribute to the beneficial effects of sitagliptin not only on β-cells but also on atherosclerosis after long-term treatment.

## Conclusion

This is the first report of a direct comparison between sitagliptin and glimepiride and their effects on endothelial cell function. Our results show that sitagliptin has a favorable anti-inflammatory effect and also positive effects on lipid metabolism compared with glimepiride, while effects on endothelial function did not differ between groups.

## Limitation

A potential limitation of our study is that it was not blinded. To deal with this limitation, the investigator who performed the FMD was unaware of the patient status and medical history. We consider that, to resolve this potential issue completely, our findings need to be validated in a double-blind study. Additional limitations include the half-time of sitagliptin. The last dose of sitagliptin was administered one day prior to the assessment of FMD. The t_max_ and elimination half-life of sitagliptin are reported to be 2 to 5 hours and 9.6 to 11.6 hours, respectively. Therefore, the plasma concentrations of sitagliptin might have been too low to have a direct effect on FMD. Finally, we were not able to obtain the data of the second FMD examination in nearly 10% of the participants for the reasons mentioned above. To resolve this issue, we employed multiple imputations for the missing data.

## Supporting Information

S1 Consort Checklist CONSORT 2010 checklist. (DOCX)

S1 Protocol Protocol of SAIS-1 Study. (DOCX)

S2 Protocol Protocol of SAIS-1 Study in Japanese. (DOC)

S1 Table All raw data in this study at week 0. (XLSX)

S2 Table All raw data in this study at week 26. (XLSX)

S3 Table Relationship between baseline BMI and changes in %FMD and metabolic parameters pre- and post-treatment with sitagliptin. (TIF)
