# Supplementary material for: A Randomized Controlled Trial Comparing the Effects of Sitagliptin and Glimepiride on Endothelial Function and Metabolic Parameters: Sapporo Athero-Incretin Study 1 (SAIS1)
Source: PLoS One. 2016 Oct 6;11(10):e0164255. doi: 10.1371/journal.pone.0164255 (PMC5053511; doi:10.1371/journal.pone.0164255)
Supplement: S2 Protocol — (DOC) [file pone.0164255.s003.doc]

自主臨床研究

**2型糖尿病患者におけるシタグリプチンの**

**血管内皮機能・代謝に与える影響の研究**

**研　究　実　施　計　画　書**

－グリメピリドを対照薬とした前向き無作為割付非盲検並行群間比較研究－

研究責任者

三好　秀明　（北海道大学病院　内科Ⅱ）

研究分担者

古本 智夫 (北海道大学病院　循環器内科)

辻野 一三 (北海道大学病院　内科Ⅰ)

近藤 琢磨 (北海道大学病院 内科Ⅱ)

永井 聡 (北海道大学病院　内科Ⅱ)

渡部　拓 (北海道大学病院　内科Ⅰ)

中村　昭伸　(北海道大学病院　内科Ⅱ）

曺 圭龍 (北海道大学病院　内科Ⅱ)

亀田 啓 (北海道大学病院　内科Ⅱ)

野本 博司 (北海道大学病院　内科Ⅱ)

三次 有奈 (北海道大学病院　内科Ⅱ)

山本 浩平 (北海道大学病院　内科Ⅱ)

山本 知穂 (北海道大学病院　内科Ⅱ)

小梁川直秀 (北海道大学病院　内科Ⅱ)

宮　愛香　 (北海道大学病院　内科Ⅱ)

一山　芽衣 (北海道大学病院　内科Ⅱ)

高野　善成 (北海道大学病院　内科Ⅱ)

北尾　直之 (北海道大学病院　内科Ⅱ)

作成日

2013年1月25日　計画書案　第 7版作成

2013年3月12日　計画書案　第 8版作成

2013年4月 1日　計画書案　第 9版作成

2013年6月 5日　計画書案　第10版作成

**1．研究の背景**

糖尿病は動脈硬化性疾患の危険因子の一つであり、我が国の疫学データからも非糖尿病患者と比較し冠動脈疾患の発症率が2.6倍に上昇すると報告されている^1)^。また、境界型の段階においてもリスクの増大を認め、腹部肥満を基盤とし耐糖能異常や高血圧、脂質代謝異常のうち複数を合併するメタボリックシンドロームや喫煙例ではリスクはさらに増大するため、これらの危険因子を包括的にコントロールすることが動脈硬化性疾患の管理に重要である^2)^。脂質異常症に用いられるスタチン系薬剤においては心血管イベント抑制効果が認められるが、これは単にLDLコレステロールを低下させることのみによるのではなく、抗炎症作用・血管内皮機能改善作用など’pleiotropic effect’と呼ばれる作用が関与しているとされる^3)^。糖尿病治療薬であるチアゾリジン誘導体や降圧薬であるアンジオテンシンⅡ受容体拮抗薬などでも同様の作用が報告されており、リスクを有する患者の治療において有用とされている。

近年本邦において、小腸から分泌されるインスリン分泌を促進する物質であるインクレチンの分解酵素であるDipeptidyl peptidase-4(以下DPP-4)を阻害することにより、血糖コントロールの改善を図る薬剤であるDPP-4阻害薬が、実地臨床の場で使用可能となった。優れた血糖降下作用はもとより、インクレチンには臨床治験段階から血圧降下、心筋保護^4)^、心不全抑制作用^5)^が報告されており、上記薬剤による抗動脈硬化への影響は興味が持たれる点である。2型糖尿病患者の治療については、マニュアル上、生活習慣の改善によっても血糖コントロール不十分な際には、SU薬もしくはビグアナイド薬から投薬開始することが推奨されているが^6)^、DPP-4阻害薬は新薬であり臨床的エビデンスが十分でないとの理由から外されている^6)^。しかし、DPP-4阻害薬が有する優れた血糖降下作用や低血糖を起こさない、体重増加を来たさないといったメリットから、第一選択薬として推奨している専門医も多い。さらにこの薬剤が有する可能性がある抗動脈硬化や膵β細胞保護効果が明らかになった際には、2型糖尿病の治療コンセプト、DPP-4阻害薬のポジショニングが変貌する可能性もある。

　一方、動脈硬化性疾患の評価法として、以前より日常診療において用いられていた頚部血管エコーやPWVなどの方法のほかに、血管内皮機能の指標としてFlow Mediated Dilatation(以下、FMD)が提唱され、近年のガイドラインにも取り入れ始められており^7)^、今後の更なる普及が待たれるとことである。チアゾリジン誘導体やα-グルコシダーゼ阻害薬でのFMDを利用した血管内皮機能改善効果を論じた報告も認められており^8)^、DPP-4阻害薬が上記検査などを用いた血管内皮機能の評価や代謝にどのような影響を及ぼしているのかを明らかにすることは臨床的にも重要であると考える。

　今回2型糖尿病患者に対し、血糖降下作用に優れるスルホニルウレア薬(以下、SU薬)であるグリメピリドと、DPP-4阻害薬であるシタグリプチンとを無作為化して投与し、血管内皮機能、β細胞機能および各種バイオマーカーの変化を比較することは意義があると考え、実施する。

**2．研究の目的**

2型糖尿病罹患患者において、SU薬を追加投与した群、DPP-4阻害薬を追加投与した群とで動脈硬化・バイオマーカーへの影響を、多施設共同・無作為・非盲検・比較試験にて検討する。

**3．試験薬の概要**

3.1　アマリール®0.5mg錠・アマリール®1mg錠

・一般名：グリメピリド

・劇薬、処方せん医薬品、薬価収載(2010年5月(0.5mg錠)・2000年4月(1mg錠))

・製造・販売元：サノフィ・アベンティス株式会社

・作用機序：主に膵β細胞の刺激による内因性インスリン分泌の促進により、血糖降下作用 を発現する。

・適応症：2型糖尿病(ただし、食事・運動療法のみで十分な効果が得られない場合に限る)

・用法・用量：グリメピリドとして1日0.5mg～1mgより開始し、適宜増減する。

・有害事象：955例中158例(16.54%：臨床検査値異常も含む)。低血糖は39例(4.08%)で、臨 床検査値上の異常は、AST, ALT, γ-GTP, LDHの上昇など。

・臨床使用成績：1～4mg投与時、改善率(HbA1c 1.0%以上低下した症例)：67.6%・HbA1c： -1.32%(プラセボ群は+0.16%)

・薬価：　0.5mg錠13.1円・1mg錠22.3円

・その他：アマリール®0.5mg錠・アマリール®1mg錠の添付文書を参照

3.2　ジャヌビア®50mg錠　ないしグラクティブ®50mg錠

・一般名：シタグリプチン

・処方せん医薬品、薬価収載(2009年12月)

・製造・販売元：MSD株式会社、小野薬品工業株式会社

・作用機序：インクレチン分解酵素であるDPP-4を高選択的に阻害し、活性型インクレチン 濃度を上昇させ、血糖依存的に強力な血糖降下作用を示す。

・適応症：2型糖尿病(ただし、食事・運動療法やそれらに加えスルホニルウレア剤、ビグア ナイド系薬剤、チアゾリジン系薬剤による治療で十分な効果が得られない場合)

・用法・用量：シタグリプチンとして50mgを1日1回経口投与

・有害事象：1190例中96例(8.1%)。主なものは低血糖、便秘など。臨床検査値上の異常変動 は、1188例中49例(4.1%)に認められ、主なものはAST, ALT, γ-GTPの上昇など。

・臨床使用成績：50mg投与時、HbA1c：-1.0%・空腹時血糖：-18mg/dl・食後2時間値：-52mg/dl

・薬価：50mg錠179.3円

・その他：ジャヌビア®50mg錠ならびにグラクティブ®50mg錠の添付文書を参照

**4．対象患者および適格性の基準**

（1）対象患者のうち、（2）選択基準をすべて満たし、かつ（3）除外基準のいずれにも該当しない場合を適格とする。

（1）対象患者

北海道大学病院内科Ⅱ、内科Ⅰまたは本研究に参加する施設に通院中の2型糖尿病患者を対象とする。

（2）選択基準

①同意取得時において年齢が20歳以上の患者

②同意取得時においてHbA1c6.5～8.0%(JDS値)の患者

③前治療として食事・運動療法のみ、あるいはビグアナイド系薬剤を内服している患者(グリニド系薬剤・αグルコシダーゼ阻害薬を内服中の患者に関しては、同薬の投与中止後1ヶ月たっていれば参加可能とする)

④血圧と脂質のコントロールが安定していて、期間中薬物の追加が必要ないと予想される患者

⑤本研究への参加にあたり十分な説明を受けた後、十分な理解の上、患者本人の自由意思による文書同意が得られた患者

（3）除外基準

①インスリンにて加療中の患者

②重篤な肝疾患、腎疾患、心疾患の合併により、本研究への参加が困難な患者

③狭心症、心筋梗塞、脳梗塞、閉塞性動脈硬化症の既往のある患者

④降圧薬を4剤以上内服している患者

⑤脂質治療改善薬を3剤以上内服している患者

⑥本試験で使用する薬剤の成分に対し、過敏症の既往のある患者

⑦妊婦、授乳婦または妊娠している可能性のある患者

⑧その他、研究責任者が被験者として不適当と判断した患者

**5．研究の方法**

（1）研究の種類・デザイン

多施設共同、無作為化非盲検並行群間比較研究

（2）試験のアウトライン

①　対象となる被験者に対して各施設での同意取得後に、北海道大学病院循環器内科にて0週の検査を行う。その際、前治療としてグリニド系薬剤・αグルコシダーゼ阻害薬の投与が行われていた場合は、組み入れまでに1ヶ月以上の休薬期間を設けることとする。

②　0週の検査が終了後、投薬開始までの期間は1ヶ月以内とする。無作為に2群の振り分けを行なった後、各施設でグリメピリド0.5mg～2mg/日またはシタグリプチン50～100mg/日を投与を開始する。

③　24週までの血糖コントロールは同意を取得した各施設で4～8週ごとに行っていく。

④　24週で再び北海道大学病院循環器内科にて検査を行い、試験を終了する。

| 登録時 | 0週 | | 4～8週毎 | 24週 |
| --- | --- | --- | --- | --- |
| 同意書取得・患者背景 | 無作為割付・投与前検査 | |  | 終了時検査 |
| 食事・運動療法  あるいは  メトホルミン単剤 | グリメピリド  追加群 | 0.5mg  ～2mg | 0.5mg  ～2mg | 0.5mg  ～2mg |
|  | シタグリプチン  追加群 | 50mg～100mg | 50mg～  100mg | 50～  100mg |
| FMD/Endo-PAT | ● |  |  | ● |
| TBI | ● |  |  | ● |
| タスクフォースモニター | ● |  |  | ● |
| 血糖(HbA1c、FPG) | ● |  | ● | ● |
| バイオマーカー測定 | ● |  |  | ● |
| 一般臨床検査 | ● |  |  | ● |

※上記の他、服薬状況・副作用確認は来院ごとに各施設で確認することとする。

●は必須項目

（3）試験薬の投与方法

アマリール®0.5mg錠(商品名)(一般名　グリメピリド)ないし1.0mg錠を0.5mg/日～2mg/日、またはジャヌビア®50mg錠ないしグラクティブ®50mg錠(商品名)(一般名　シタグリプチン)を50～100mg/日を投与する。いずれの群でもHbA1c 6.5%未満を目標として、主治医判断にて上記範囲内で投与量の調節を行うこととする。メトフォルミンの追加投与は可能とするが、メトフォルミン以外の経口血糖降下薬の追加投与は行わず、食事運動療法の強化など行い目標達成を目指す。

（4）併用薬（療法）についての規定

期間中に降圧薬や脂質異常治療薬が追加投与された場合には脱落とする。

（5）症例登録、割付の方法

①症例登録

　症例登録・報告書を研究事務局に送付する。

②試験薬の割り付け

　北海道大学病院初診時に行った検査結果が研究事務局に送られ、割付表に従いグリメピリド投与群、シタグリプチン投与群のいずれかに無作為に割り付けが行われる。

（6）被験者の研究参加予定期間

各被験者は同意後、北海道大学病院で検査を受け、割り付けられた薬剤について各施設で投薬開始され、24週間の観察期間で参加する。

（7）研究終了後の対応

本研究終了後は、この研究で得られた成果も含めて、研究責任者は被験者に対し最も適切と考える医療を提供する。

**6．観察および検査項目**

以下の項目について、観察および検査を実施し、そのデータを本研究に利用する。これらのうち、④⑤⑥ならびに下線を引いた項目は本研究のために実施するものである。

①患者背景：患者イニシャル、識別コード、年齢、性別、診断名、身長、体重、BMI、腹囲、罹病期間、合併症、喫煙・飲酒の有無、薬剤服薬内容

②外来血圧、脈拍数

③臨床検査（空腹時）

尿検査（尿糖、尿蛋白(定性)、尿中アルブミン/クレアチニン比）、血糖(HbA1c、空腹血糖)、インスリン、プロインスリン、空腹時血清脂質(総コレステロール、中性脂肪、HDLコレステロール、LDLコレステロール、FFA、アポ蛋白、RLP-C)、AST、ALT、γ-GTP、尿酸、BUN、血清クレアチニン、Na、K、Cl、hs-CRP、NT-proBNP

バイオマーカー：アディポネクチン、PAI-1、TNF-α、SOD、尿中8-OHdGなど動脈硬化関連マーカー

　尚、バイオマーカー測定用として、0週時および24週時に1回あたり10 mlの追加採血をおこなう。被験者の同意が得られた場合は、測定後の残余検体を保存し、将来、新しいバイオマーカー等がみつかった場合に追加測定をする可能性がある。

④Toe brachial index(TBI)

⑤Flow mediated dilatation(FMD) /Endo-PAT：FMD/Endo-PATは血糖レベルや測定時間により変動する。ゆえにFMD/Endo-PATは空腹で午前中の施行とする。実施に際してはガイドラインに従い、施行6時間前よりカフェイン、タバコ、ビタミン剤の服用は禁止し、温度調節された部屋で実施する。但し、血管拡張薬も含め服用薬剤は継続状態とする(当日朝のみ中止し、検査終了後速やかに内服することとする)。

⑥タスクフォースモニター：非侵襲的心機能測定装置であるタスクフォースモニター(CNSystems, 日本光電)を用いて、心拍出量、末梢血管抵抗、自律神経機能などの評価を行う。具体的には心電図を装着し、頸背部に張り付けた電極より微細な高周波電流を流し、心拍動により生じる胸郭のインピーダンス変化を計測し、増幅処理して波形を表示する。またその値を演算処理することで、循環動態の指標となる各種の数値を計算する。また、上腕にまいたカフにより非観血的血圧測定を、手指にとりつけたカフにより非観血的連続血圧測定を行う。⑤検査と同時に行うことより、測定に際しての条件は⑤と同様とする。

**7．予想される利益および不利益（副作用）**

（1）予想される利益

本研究で実施するFMD/Endo-PAT・タスクフォースモニターといった検査や各種バイオマーカー測定にて、その結果により患者の病態がより明確となり適切な治療の選択が可能となる。また、研究成果により将来の医療の進歩に貢献できる可能性がある。

（2）予想される不利益（副作用）

詳細は添付文書を参照。グリメピリドでは高用量での頻度の高い副作用として低血糖があり、シタグリプチンでは低血糖(約1％)と便秘が報告されている。

①血管内皮機能検査(加圧試験)について：上腕動脈を血圧計のカフで加圧することにより、疼痛が生じる可能性があるが、本検査は臨床でも広く普及しており疼痛は認容範囲内である。しかし疼痛が高度の場合、担当医師の判断で中止することもある。

②採血量について：本研究により1回あたりの採血量が10ml増加する。しかし、これらは被験者の症状や治療経過に影響を与えないものと考えられる。

**8．評価項目（エンドポイント）**

（1）主要評価項目

試験薬投与前後でのFMDの変化量を投与群間で比較する。

（2）副次的評価項目

以下の項目の試験薬投与前後の投与群間の変化量

①血糖(HbA1c、空腹時血糖)

②膵β細胞機能の評価

③TBI・タスクフォースモニターでの各種測定値の変化量

④各種血液検査値、尿検査値、バイオマーカー

⑤Endo-PAT

**9．個々の被験者における中止基準**

（1）研究中止時の対応

研究責任者または研究分担者（以下、研究担当者）は、次に挙げる理由で個々の被験者について研究継続が不可能と判断した場合には、当該被験者についての研究を中止する。その際は、必要に応じて中止の理由を被験者に説明する。また、中止後の被験者の治療については、被験者の不利益とならないよう、誠意を持って対応する。

（2）中止基準

①　被験者から研究参加の辞退の申し出や同意の撤回があった場合

②　登録後に適格性を満足しないことが判明した場合

③　原疾患の悪化のため、試験薬の継続投与が好ましくないと判断された場合

④　合併症の増悪により試験の継続が困難な場合

⑤　有害事象により試験の継続が困難な場合

⑥　妊娠が判明した場合

⑦　著しくコンプライアンス不良の場合

⑧　食事・運動療法の順守できない患者

⑨　本研究全体が中止された場合

⑩　その他の理由により、研究担当者が研究の中止が適当と判断した場合

**10．有害事象発生時の取扱い**

（1）有害事象発生時の被験者への対応

研究担当者は、有害事象を認めたときは、直ちに適切な処置を行うとともに、診療録ならびに症例報告書に記載する。また、試験薬の投与を中止した場合や、有害事象に対する治療が必要となった場合には、被験者にその旨を伝える。

（2）重篤な有害事象の報告

重篤な有害事象は、薬事法施行規則第273条に準じて次の通りに定義する。

1）死亡または死亡につながるおそれ

2）入院または入院期間の延長

3）障害または障害につながるおそれ

4）後世代または先天性の疾病または異常

研究責任者は、研究期間中の全ての重篤な有害事象、研究終了（中止）後に試験薬との関連性が疑われる重篤な有害事象について、速やかに自主臨床研究事務局を通じて病院長に報告する。報告は、自主臨床研究標準業務手順書に準じて、第一報（緊急報告）および第二報以降（詳細報告）とする。

（3）重要な有害事象の報告

該当なし。

（4）その他の有害事象

その他の有害事象については、研究担当者は適切に診療録および症例報告書に記載する。

**11．研究実施計画書等の変更**

本研究の研究実施計画書や同意説明文書の変更または改訂を行う場合は、あらかじめ自主臨床研究審査委員会の承認を必要とする。

**12．研究の変更、中止・中断、終了**

（1）研究の変更

本研究の研究実施計画書や同意説明文書の変更または改訂を行う場合は、あらかじめ北海道大学病院自主臨床研究審査委員会（以下、審査委員会）の承認を必要とする。

（2）研究の中止、中断

研究担当者は、以下の事項に該当する場合は、研究実施継続の可否を検討する。

①被験者の組み入れが困難で、予定症例数に達することが極めて困難であると判断されたとき。

②予定症例数または予定期間に達する前に、研究の目的が達成されたとき。

③審査委員会により、実施計画等の変更の指示があり、これを受入れることが困難と判断されたとき。

研究責任者は、審査委員会により中止の勧告あるいは指示があった場合は、研究を中止する。また、研究の中止または中断を決定した時は、速やかに病院長にその理由とともに文書で報告する。

（3）研究の終了

研究の終了時には、研究責任者は速やかに研究終了報告書を病院長に提出する。

**13．研究実施期間**

平成23年3月～平成26年3月31日（登録締切日：平成25年9月30日）

**14．目標症例数とその設定根拠および統計解析方法**

（1）目標症例数 1群55例、合計110例

【設定根拠】

シタグリプチンの投与によるFMDへの影響に関しての詳細な報告は現時点で認められない。Papathanassiou K らの報告では、グリメピリドに対するピオグリタゾンの6ヶ月後のFMD変化を評価しており、グリメピリド群での変化が0.1（標準偏差＝1.0）ピオグリタゾン群での変化が2.0（標準偏差=2.0）と報告されている^9)^。本研究では、シタグリプチンにおける6ヶ月後のFMD変化量が少なくとも1.0（標準偏差=2.0）であると仮定し、グリメピリド群の変化量および標準偏差は0.1（標準偏差＝1.0）として、等分散を仮定しないt検定を行う。検出力80%、有意水準5%の下では、1群50例が必要で、通院中断などの脱落例を考慮し1群55例が必要となる。

（2）統計解析方法

薬剤投与時のFMDの変化量に関して、全被験者の治療前後の値を計測し、治療前後の平均値ならびに標準偏差を示す。主要な解析では、2群間の登録時から6ヶ月後のFMD変化量について等分散を仮定しないt検定を用いて比較する。また、各々の変化量についても多重比較検定により比較を行う。その他、各種バイオマーカーやβ細胞機能の変化を表すパラメーターについても同様に全被験者の変化を求め、探索的に群間の違いならびにパラメーター間の関連を検討する。

**15．被験者の人権に対する配慮および個人情報の保護の方法**

本研究のすべての担当者は、「ヘルシンキ宣言（2008年10月修正）」および「臨床研究に関する倫理指針（平成20年7月31日改正、以下臨床研究倫理指針）」を遵守して実施する。

研究実施に係る試料等を取扱う際は、被験者の個人情報とは無関係の番号を付して管理し、被験者の秘密保護に十分配慮する。試料等を研究事務局等の関連機関に送付する場合はこの番号を使用し、被験者の個人情報が院外に漏れないよう十分配慮する。また、研究の結果を公表する際は、被験者を特定できる情報を含まないようにする。研究の目的以外に、研究で得られた被験者の試料等を使用しない。

**16．同意取得方法**

研究担当者は、審査委員会で承認の得られた同意説明文書を被験者に渡し、文書および口頭による十分な説明を行い、被験者の自由意思による同意を文書で取得する。

研究担当者は、被験者の同意に影響を及ぼす情報が得られたときや、被験者の同意に影響を及ぼすような実施計画等の変更が行われるときは、速やかに被験者に情報提供し、研究に参加するか否かについて被験者の意思を予め確認するとともに、事前に審査委員会の承認を得て同意説明文書等の改訂を行い、被験者の再同意を得ることとする。

同意説明文書には、以下の内容を含むものとする。

①研究への参加は任意であること、同意しなくても不利益を受けないこと、同意は撤回できること

②研究の意義（背景）、目的、対象、方法、実施期間、予定被験者数

③研究に参加することにより期待される利益、起こりえる不利益

④個人情報を含めた試料等の取扱い、保存期間と廃棄方法、研究方法等の閲覧

⑤研究成果の発表および特許が発生した場合の取扱い

⑥研究に係る被験者の費用負担、研究資金源と利益相反

⑦研究の組織体制、研究に関する問い合わせ、苦情等の相談窓口（連絡先）

⑧被験者に健康被害が発生した場合の対応と補償の有無

**17．被験者の健康被害への対応と補償**

本研究の実施に伴い、被験者に健康被害が発生した場合は、研究担当者は適切な処置を講じる。

また、健康被害に対する補償は、臨床研究倫理指針に従って行う。すなわち、死亡および重度障害（一級および二級）に対し、補償金を準備する。これ以外の健康被害に対しては、被験者の保険診療内で検査や治療等、必要な処置を行う。

**18．被験者の費用負担**

　本研究のうち、動脈硬化やバイオマーカーの測定など研究目的で実施する血液内皮機能の各種検査に係る費用のすべては研究責任者が所属する診療科の研究費で賄う。病院受診料とHbA1c採血検査については通常の保険診療内で行われるため、研究に参加することによる被験者の費用負担は発生しない。

　なお、被験者の負担軽減を目的として、動脈硬化検査(FMD Endo-PAT、TBI、タスクフォースモニター)施行のため当院を受診の際に、1回につきクオカードで4,000円分を支給することとし、このことを被験者に十分説明した上で、研究への参加の判断を求める。なお、来院したがFMDなどの検査を施行し得なかった場合(喫煙・食事など、FMD Endo-PATの施行条件を満たせていない場合)においては、支給は行わないこととする。

**19．記録の保存と研究結果の公表**

研究責任者は、研究等の実施に係わる重要な文書（申請書類の控え、病院長からの通知文書、各種申請書・報告書の控、同意書、その他データの信頼性を保証するのに必要な書類または記録等）を、研究の中止または終了後3年が経過した日までの間保存し、その後は個人情報に注意して廃棄する。

研究担当者は、本研究の成果を関連学会等において発表することにより公表する。

**20．研究資金および利益相反**

本研究は、研究責任者が所属する診療科の研究費で実施する。また、利益相反審査の取り扱いは、各施設の規定に従って実施する。北海道大学病院の研究担当者は、「北海道大学病院における臨床研究に係る利益相反マネジメント内規」の規定にしたがって、利益相反審査委員会に必要事項を申告し、その審査と承認を得るものとする。

**21．研究実施体制**

本研究は以下の体制で実施する。

【研究責任者】

- 三好　秀明　 （北海道大学病院　内科Ⅱ・講師）　 011-706-5915

【研究分担者】

古本 智夫 (北海道大学病院 循環器内科・非常勤講師) 011-706-6974

辻野 一三 (北海道大学病院 内科I ・講師) 011-706-5911

近藤 琢磨 (北海道大学病院 内科Ⅱ・助教) 011-706-5916

永井 聡 (北海道大学病院　内科Ⅱ・客員臨床講師) 011-706-5915

渡部　拓 (北海道大学病院　内科I・助教) 011-706-5911

中村　昭伸 (北海道大学病院 内科Ⅱ・助教) 011-706-5916

曺 圭龍　 (北海道大学病院 内科Ⅱ・特任助教) 011-706-5916

亀田 啓 (北海道大学病院　内科Ⅱ・医員) 011-706-5916

野本 博司　 (北海道大学病院 内科Ⅱ・医員) 011-706-5916

三次 有奈　 (北海道大学病院　内科Ⅱ・医員) 011-706-5916

山本 浩平　 (北海道大学病院 内科Ⅱ・医員) 011-706-5916

山本 知穂 (北海道大学病院 内科Ⅱ・医員) 011-706-5916

小梁川 直秀 (北海道大学病院 内科Ⅱ・医員) 011-706-5916

宮　愛香　　　　(北海道大学病院　内科Ⅱ・医員) 011-706-5916

一山　芽衣 　　(北海道大学病院　内科Ⅱ・医員) 011-706-5916

高野　善成 　　(北海道大学病院　内科Ⅱ・医員) 011-706-5916

北尾　直之 　　(北海道大学病院　内科Ⅱ・医員) 011-706-5916

　○研究代表者

【研究事務局】

北海道大学病院内科Ⅱ　責任者：三好　秀明（北海道大学病院　内科Ⅱ・講師）

連絡先：〒060-0838 北海道札幌市北区北15条西7丁目

北海道大学病院　内科Ⅱ　医局　011-706-5915

【症例割付実施機関】

北海道大学病院 高度先進医療支援センター　データ管理部門

　　　責任者：大庭　幸治（北海道大学病院高度先進医療支援センター・助教）

住　所：〒060-8648 北海道札幌市北区北14条西5丁目

電　話：011-706-7413

【参加施設】　五十音順

青木内科クリニック、大塚眼科、大通り内科クリニック、小笠原クリニック札幌病院附属外来プラザ、岡本内科クリニック、小野百合内科クリニック、クラーク病院、栗原内科、国立病院機構北海道医療センター、佐々木内科、札幌社会保険総合病院、市立札幌病院、桑園中央病院、時計台記念病院、斗南病院、北海道社会保険病院、北海道中央労災病院せき損センター、北陵内科、萬田記念病院、NTT東日本札幌病院

**22．参考資料・文献リスト**

1) Fujishima M et al: Diabetes, 45(Suppl3), S14-S16

2) 動脈硬化性疾患ガイドライン 2007, 日本動脈硬化学会

3) 脂質異常症治療ガイド 2008年版, 日本動脈硬化学会

4) Sokos GG et al: Journal of Cardiac Failure. 2006; 12: 694-699

5) Nikolaidis LA et al: Circulation. 2004; 109: 962-965

6）国立国際医療研究センター病院　糖尿病標準診療マニュアル　2010

7) 高血圧症ガイドライン2009, 日本高血圧学会

8) Fabrice M. A. C. Martens et al: Journal of Cardiovascular Pharmacology. 2005; 46: 773-778

9) Papathanassiou K, Naka KK, Kazakos N, et al. Pioglitazone vs glimepiride: Differential effects on vascular endothelial function in patients with type 2 diabetes. Atherosclerosis 2009; 205: 221-6.
