# Supplementary figures and images for: A Randomized Controlled Trial Comparing the Effects of Sitagliptin and Glimepiride on Endothelial Function and Metabolic Parameters: Sapporo Athero-Incretin Study 1 (SAIS1)
Source: PLoS One. 2016 Oct 6;11(10):e0164255. doi: 10.1371/journal.pone.0164255 (PMC5053511; doi:10.1371/journal.pone.0164255)

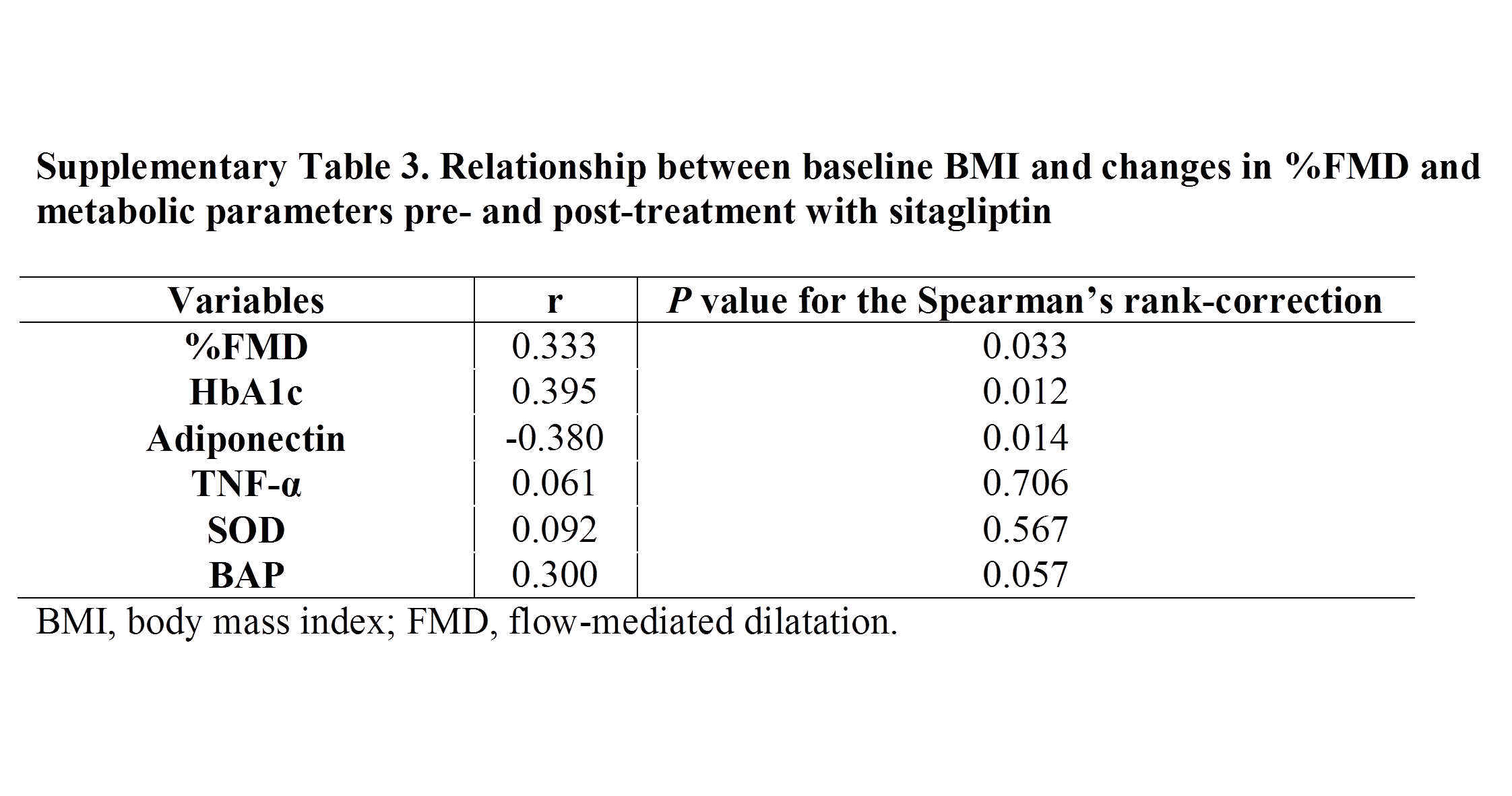

Supplement: S3 Table — (TIF) [file pone.0164255.s006.tif]
